# Pathogenesis of rabies in a pregnant HIV immune-compromised woman in Zambia: A case report

**DOI:** 10.4102/jphia.v16i1.1456

**Published:** 2025-09-29

**Authors:** Martin Nyahoda, Mukatimui K. Munalula, Agripa Lungu, Walter Muleya, Selia Ng’anjo, Willies Silwimba, Chrispin Mwando, Joyce N. Shampile

**Affiliations:** 1 Department of Disease Control, School of Veterinary Medicine, University of Zambia, Lusaka, Zambia; 2 University Teaching Hospitals, Women and Newborn Hospital, Lusaka, Zambia

**Keywords:** rabies, pathogenesis, HIV, pregnant, Zambia

## Abstract

Rabies is a fatal neglected tropical zoonotic disease caused by neurotropic viruses of the genus Lyssavirus in the family Rhabdoviridae. We report the disease progression in a 30-year-old woman, in her eighth pregnancy, living with human immunodeficiency virus (HIV) on antiretroviral therapy (ART), who presented with neurological symptoms including aggression, restlessness, fever and vomiting 20 days following rabies exposure through multiple dog bites on the face and upper limbs. She had received a 4-dose regimen of rabies post-exposure prophylaxis (PEP), starting 2 days after exposure, with subsequent doses given 3 and 7 days later, while the 4th dose was administered 20 days after exposure. Wound washing was not performed, and rabies immunoglobulin was not administered as recommended by the World Health Organization for category 3 exposures. The disease rapidly progressed to rabies encephalitis, leading to death within 6 days of admission. Reverse transcriptase polymerase chain reaction (RT-PCR) performed on cerebral spinal fluid (*n* = 3) and nasopharyngeal swabs (*n* = 2) confirmed the diagnosis of rabies infection. Although the incubation period and symptomatology did not significantly deviate from documented classical cases, a compromised immunity evidenced by a low cluster of differentiation 4 (CD4) T-cell count of 382, coupled with non-adherence to recommended best practices for wound management and PEP administration, may have influenced the rapid disease progression. This case reveals the need for capacity building in health workers and the community to improve knowledge of rabies post-exposure response in Africa.

## Background

Rabies is a viral infectious zoonotic disease which causes a progressive and fatal inflammation of the brain and the spinal cord.^1,2^ The disease is caused by neurotropic lyssaviruses which include rabies virus (RABV), a bullet-shaped negative single-strand ribonucleic acid (RNA) virus of the family Rhabdoviridae.^1,2^ The genome encodes five major proteins which are the nucleocapsid protein (N), phosphoprotein (P), glycoprotein (G), the matrix protein (M) and the RNA dependent polymerase (L).^3,4^ Global mortality estimates attributable to rabies infection may be over 59000 annually in over 150 countries with rural populations accounting for the highest burden.^1,5^ The estimated global rabies mortality translates to over 160 persons dying from rabies infection each day. Challenges in laboratory testing, sporadic epidemiological surveillance and unreported cases have possibly resulted in underestimating the burden of disease and mortality attributable to rabies.^1,5,6^ The majority of deaths from rabies (95%) occur in Africa and Asia.^5,7^ Globally, the burden of rabies infection is estimated to cost about $583.5 million annually with livestock losses in Africa and Asia reaching an estimated $123 million.^4^ In Zambia, there is an estimated 15000 dog bites resulting in 50 deaths because of rabies annually.^8^ The incubation period of rabies varies, but may typically range from 1 week to over 1 year.^9^ Symptoms may include aggressiveness, restlessness, myalgia, hydrophobia and confusion.^10,11^

We report the disease progression in a 30-year-old pregnant woman living with human immunodeficiency virus (HIV), a gravida 8 parity 7 at 24 weeks 3 days gestation from a rural district who was referred to University Teaching Hospitals–Women and Newborn Hospital on 11 February 2025 for further management of suspected rabies in pregnancy. The patient was living with HIV on antiretroviral therapy (ART) with a low cluster of differentiation 4 (CD4) T cell count of 382. Studies have shown that CD4 T cell immune suppression may result in increased viral replication, lower immunoglobulin G (IgG) antibody production and lower CD4 T cell-derived cytokines leading to rapid disease progression.^12^ She had a history of being bitten by an unvaccinated dog on multiple body sites including the face and upper limbs on 19 January 2025. She received the 1st dose of rabies post exposure prophylaxis (PEP) on 21 January 2025, the second and third doses after 3 days and 7 days respectively, while the 4th dose was administered on 10 February 2025. No wound washing or administration of rabies immunoglobulin as recommended was performed. She also received ceftriaxone, metronidazole, metoclopramide, dexamethasone and atropine from the referring lower-level facility. On admission, the vitals were taken (Table 1).

**TABLE 1:** Vital readings on admission.

Vital	Reading
Blood pressure (mmHg)	96/52
Peripheral oxygen saturation (SpO_2_) (%)	96–98
Respiration (breaths per minute)	26
Pulse (beats per minute)	112
Temperature (°C)	37.6

## Case presentation

On admission, the woman presented with neurological symptoms, restlessness, aggression and vomiting. She also had neck pain and stiffness, difficulties in breathing and was not able to speak. However, the patient did not present with photophobia.

Obstetric ultrasound scan performed on admission revealed a live singleton, intrauterine foetus in cephalic presentation at 24 weeks gestation. Foetal heart rate was 156 beats/min. The placenta was posterior and not low lying, while the cervical ostium [*ostium internum uteri*] was closed. No obvious foetal anomalies were observed.

Investigations revealed an elevated lactate dehydrogenase, leukocytosis indicative of encephalitis and response to infection respectively with a low CD4 count (Table 2). The patient became hydrophobic after seeing water. She also had excessive secretions. She developed tachypnea and tachycardia with tongue and lip swelling. She was isolated in a dark room and kept away from water. She was prescribed ceftriaxone 1 g intravenous (IV), metronidazole 500 mg IV, diazepam 5 mg IV and metoclopramide 2 mg IV.

**TABLE 2:** Complete blood count and biochemistry results.

Test	Test values	Reference interval	Flags
**Liver function tests**			
Bilirubin unconjugated	5 µmol/L	0.0–7.0	-
Bilirubin direct	0.87 µmol/L	0.0–3.4	-
Bilirubin total	5.4 µmol/L	5.0–21.0	-
Lactate dehydrogenase	388 IU/L	135.0–246.0	High
Alkaline phosphatase	98.1 U/L	35.0–104.0	-
g-Glutamyl transferase	12.3 U/L	0.0–39.0	-
Alanine transaminase	15.1 U/L	0.0–34.0	-
Aspartate transaminase	36.5 U/L	0.0–31.0	High
Total protein	80.0 g/L	60.0–78.0	High
Albumin	33.3 g/L	35.0–52.0	Low
**Full blood count and platelets**			
White cells	16.25 × 10^9^/L	4.0–10.0	High
Red cells	4.83 × 10^12^/L	3.8–4.8	High
Haemoglobin	14.0 g/dL	12.1–16.3	-
Haematocrit level	42.4%	36.0–46.0	-
Mean corpuscular volume	87.8 fl	79.1–98.9	-
Mean corpuscular haemoglobin	29.0 pg	27.0–32.0	-
Mean corpuscular haemoglobin concentration	33.0 g/dL	32.0–36.0	-
Platelets	335 × 10^12^/L	150.0–400.0	-
Mean platelet volume	10.1 fl	8.0–10.0	-
Platelet width distribution	10.8 fl	14.0–17.0	Low
**Urea, electrolytes and creatinine**			
Sodium	134 mmol/L	136.0–145.0	Low
Potassium	4.69 mmol/L	3.5–5.1	-
Chloride	99.3 mmol/L	98.0–107.0	-
Urea	4.69 mmol/L	2.8–7.1	-
Creatinine	72.4 µmol/L	45.0–84.0	-
**Differential count**			
Neutrophils	87.1% 14.15 × 10^9^/L	2.0–7.0	High
Lymphocytes	7.1% 1.15 × 10^9^/L	1.0–3.0	-
Monocytes	5.6% 0.91 × 10^9^/L	0.2–1.0	-
Eosinophils	0.0% 0.00 × 10^9^/L	0.02–0.50	Low
Basophils	0.2% 0.03 × 10^9^/L	0.02–0.10	-
**CD4 test**			
CD4 absolute count	382 cells/µL	410–1590	Low
**Blood parasites**			
Malaria	No malaria parasites seen	-	-

CD4, cluster of differentiation 4.

The pulse was 108 beats/min (bpm) with respiration of 26 breaths per minute. She was placed on oxygen via nasal prongs and nursed in a propped up position. In order to protect the airway, the patient was intubated. Midazolam 5 mg IV and morphine 5 mg IV were administered to sedate the patient. In order to maintain her in a sedated state, midazolam 1 mg and morphine 2 mg were given hourly. She was also continued on ceftriaxone and metronidazole.

On the second day, she was diagnosed with impending respiratory failure. Ventilatory support was continued with regular suctioning of the endotracheal tube (ETT) to remove excess fluids. She became febrile with a temperature of 40 °C and a supple neck. Cerebral spinal fluid (CSF) and nasopharyngeal (NP) swabs were collected and taken to the University of Zambia, School of Veterinary Medicine, for rabies diagnosis. She was continued on ceftriaxone and respiratory support.

On the third day, the blood pressure (BP) was normal with 115/85 mmHg, but later increased to 142/106 mmHg with a pulse of 95 bpm. The patient continued to have a high fever with her temperature reaching as high as 40.8 ^o^C. Consciousness, as monitored using the Glasgow Comma Scale (GCS), dropped to 3T/15 (E 1, V 1T, M 1). Midazolam was withdrawn because of the deteriorating neurological status, while regular suctioning of the ETT was continued. The pupils were dilated with a sluggish reaction to light. The patient was maintained on 2.5 L fluids 24 h. The foetus was still live at 24 weeks 4 days.

On the fourth day, the neurological status further deteriorated, the pupils were fixed without response to light and corneal and vestibulo-ocular reflex (VOR) were absent. She continued to have tachycardia and tachypnea. Systolic BP rose to 150 mmHg while diastolic BP was 80 mmHg with a pulse of 139 bpm and peripheral oxygen (SpO_2_) saturating at 99%. The patient continued to have a sustained fever with a body temperature of 40.8 °C. She developed facial oedema and a nasal discharge which was not investigated. The general condition became critical, and ventilatory support was continued. Real-time polymerase chain reaction (PCR) performed on CSF and NP swabs using primers and the protocol designed by De Benedicts et al. (2011) detected rabies nucleoprotein (N) gene.^8^ Rabies encephalitis, paralytic stage with brain stem death was diagnosed and confirmed both clinically and using molecular methods. The foetus was still live at 24 weeks 5 days with a foetal heart rate of 153 bpm and an estimated foetal weight of 651 g. Relatives were counselled on the prognosis, and the patient was continued on supportive care.

On day five of admission, the patient remained in a critical state. She was continued with supportive care on ventilatory support. She suffered cardiac arrest at about 20:54, and no resuscitation was attempted. On review at 20:59, the pupils were fixed and dilated. No cardiopulmonary activity was detected. The patient was certified dead, relatives were informed and the body was transferred to the morgue. The patient died undelivered. The cause of death was assigned as cardiopulmonary failure due to rabies encephalitis, because of canine rabies infection with other complicating conditions being HIV retroviral disease.

## Discussion

We report the pathogenesis of rabies in a 30-year-old pregnant woman with HIV who had category 3 rabies exposure after sustaining multiple transdermal dog bites including the face and upper limbs.^13,14^ Although the incubation period for rabies may vary from 1 week to 1 year, it typically ranges between 2–3 months.^7,14^ In this case, the patient presented with rabies symptoms 20 days after exposure and died within 6 days after onset of symptoms which was 26 days after exposure. Although the incubation period for this case does not remarkably deviate from other cases, it was relatively shorter than typical cases.^7,14,15^ It is possible that the patient’s immune-suppressed status evidenced by a low CD4 count may have contributed to the rapid disease progression even though the evaluation of the antibody titre was not performed to determine the immune response to rabies PEP.^7,14,15,16^ In addition, the decrease in T cells and reduced cytokine secretions associated with the first and second trimester of pregnancy may have contributed to disease progression.^17^ The disease progression in this case further underscores the fact that although PEP is important for prevention of rabies, persons with immune suppression can develop the disease even after administration of PEP and especially in instances of poor adherence to best practices as other studies have shown.^7,18,19^ For example, a 6-year-old HIV immune compromised person in Thailand failed to respond to two regimens of pre-exposure vaccine given intramuscularly on days 0, 7 and 28 and intradermal PEP administered on days 0, 3, 7, 30 and 90.^18^ The neutralising antibodies could not reach the recommended > 0.15 IU/mL titre.

This case also demonstrates the need to adhere to best practices on post exposure wound care and PEP administration. Persons with category 3 exposure should immediately wash the wound thoroughly with soap and water including disinfection and get either a 5 or 4 dose PEP regimen with rabies immunoglobulin infiltrated around the wound as much as anatomically feasible^7,14,19^ The 5 dose regimen should be given on days 0, 3, 7, 14 and 28 while the 4 dose regimen should be administered on days 0, 3, 7, and 28.^7,14^ The lack of thorough wound washing with soap and the delay in administering PEP without rabies immunoglobulin in this case may have contributed to the rapid disease progression and its fatal outcome.^19^

The general symptomatology did not present a significant departure from typical rabies symptoms and was largely comparable to classical cases in immune-competent individuals and similar to other reported cases in immune-compromised persons with an exception of a nasal discharge.^10,11,18,19^ Generally, the patient presented with fever up to 40.8 °C, aggression, restlessness, hydrophobia and vomiting, similar to other rabies cases observed in Africa and elsewhere.^10,11,19^ Human immunodeficiency virus opportunistic infections were clinically absent. It is not clearly evident from this case that the immune suppressed status of the patient may have significantly influenced the clinical presentation of the disease. Notwithstanding, we did not perform any clinical pathology to determine the nature and characterisation of the encephalitis and neuroapoptosis in comparison with other published findings.^19^

This case provides clear evidence that rabies continues to pose a serious threat to public health especially in low-income countries. Additionally, it reveals that knowledge gaps in rabies post exposure response and management continue to exist at health facility and community level in Zambia. Although evidence from this and other cases seem to suggest that pathogenesis of rabies in immune-compromised persons does not significantly depart from that in immune competent individuals, at the very least, it demonstrates that non-adherence to best practices in administration of rabies PEP and wound management results in rapid disease progression in immune suppressed persons. However, it is important to note that we did not ascertain rabies antibody titres to determine immune response to PEP. Therefore, we recommend further investigations on rabies interactions in HIV immune compromised individuals.

Despite the fact that rabies is a fatal disease, it is preventable and can be eliminated. As studies have shown, with heightened rabies surveillance such as reporting suspected dogs to public health authorities, rabies education in communities including schools and enhanced rabies vaccination to reduce incidence in canine reservoirs, it is possible to eliminate rabies in Zambia and Africa at large.^20,21,22^
